# Integrated Electron Microscopy: Super-Duper Resolution

**DOI:** 10.1371/journal.pbio.1001639

**Published:** 2013-08-27

**Authors:** Jacomine Krijnse Locker, Sandra L. Schmid

**Affiliations:** 1 Department of Infectious Disease & Core Facility Electron Microscopy, University of Heidelberg, Heidelberg, Germany; 2 Department of Cell Biology, UT Southwestern Medical Center, Dallas, Texas, United States of America

## Abstract

Since its inception, electron microscopy (EM) has revealed that cellular membranes are organized into structurally distinct subdomains, created by localized protein and lipid assemblies to perform specific complex cellular functions. Caveolae are membrane subdomains that function as signaling platforms, endocytic carriers, sensors of membrane tension, and mechanical stress, as well as in lipid homeostasis. They were first discovered almost 60 years ago by pioneering electron microscopists. While new and exciting developments in SUPER-resolution fluorescent light microscopy facilitate studies of the spatial organization of fluorescently labeled protein components, these techniques cannot reveal the underlying cellular structures. Thus, equally exciting are developments in EM: genetically encoded probes for protein localization at sub-10 nm resolution, more powerful instruments that allow imaging of larger cell volumes, and computational methods for reconstructing three-dimensional images. Used in combination, as done by Ludwig et al. in the current issue of *PLOS Biology*, these tools reveal high-resolution insights into the composition and organization of the caveolae coat and the formation of these specialized structures. Together, these advances are contributing to a resurgence in EM.

## Abstract 2

New developments in electron microscopy used in combination reveal the elegant architecture of cellular structures at very high resolution.

## Coated Vesicles and Caveolae

**Figure 1:**
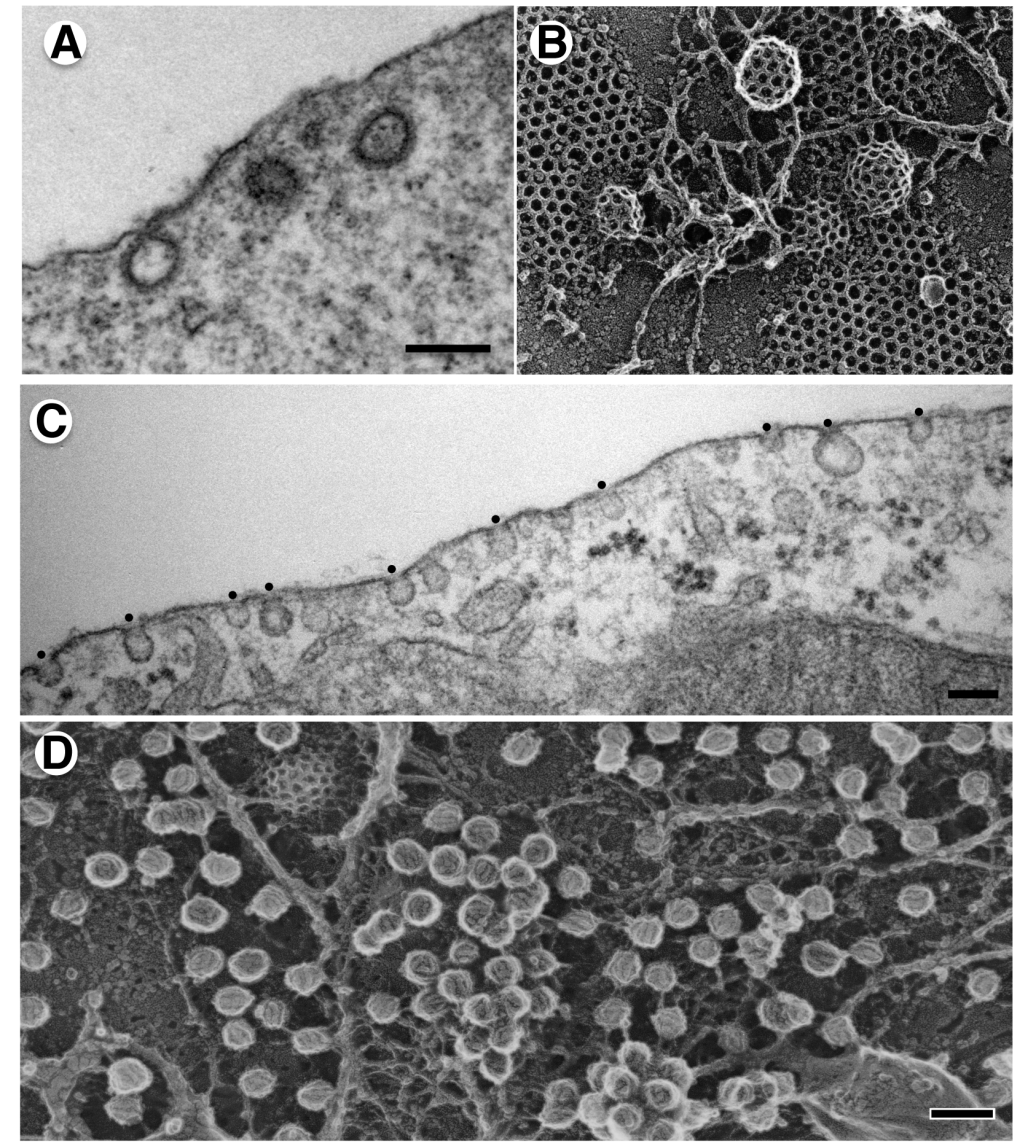
Thin section and quick-freeze deep etch images of clathrin coated vesicles and caveolae. (A) Thin section, TEMs reveal the bristled appearance of clathrin-coated pits in fixed and plastic embedded mouse fibroblasts (from [27]), bar = 200 nm. (B) Quick-freeze deep-etch images reveal the lattice-like coat on flat and rounded clathrin coated pits, courtesy of John Heuser. (C) Ultra thin section EM of caveolae in fixed and plastic embedded 3T3-L1 adipocytes (from [18]). Bar = 100 nm. (D) Deep-etch EM replica of differentiated 3T3-L1 adipocyte produced in the Heuser lab by N. Morone, iCeMS, Kyoto University, Japan. Bar = 100 nm.

Cells communicate with each other and with their environment through dynamic signaling and trafficking events that occur at their outer surface, the plasma membrane. Perhaps the best characterized mediators of trafficking from the cell surface are clathrin coated vesicles (CCVs), which are responsible for the selective internalization of nutrients, signaling receptors, and transmembrane transporters to ensure vital metabolic activities of the cell. Like many cellular structures clathrin-coated pits and vesicles were first described almost 50 years ago by electron microscopy (EM) [1]. They are decorated with a lattice-like (i.e., clathrate) coat that is easily recognized by thin section EM and beautifully revealed by quick freeze, deep-etch images of ripped off cell surfaces (Figure 1A and 1B) [2]. Because the clathrin coat is relatively stable, CCVs can be readily isolated by simple subcellular fractionation methods owing to their high density and small size. Thus, the protein composition of the coat has been defined biochemically for decades [3]–[5], and has served as the basis for identifying factors that regulate CCV assembly.

Caveolae, “little caves,” are another class of specialized internalizing structures, initially recognized in electron micrographs as smooth membrane invaginations with a characteristic bulb-like shape (Figure 1C) [6]. In addition to their role as endocytic carriers, caveolae are thought to function as static platforms for the spatial organization of signaling complexes, as mechanosensory devices, and in the regulation of lipid homeostasis (see [7] for recent review). Consistent with their diverse functions, the number and morphology of caveolae varies from tissue to tissue, and they are most abundant in endothelial cells and adipocytes. Unlike CCVs, the purification of caveolae proved to be more difficult and required the use of detergents and/or sonication [8], which can result in loss of some components and potential contamination by others. Thus, the identification of the coat components that form caveolae and regulate their function and morphology has lagged behind our understanding of CCVs.

## The Caveolae Coat

The identification of caveolin as a major component of the caveolae coat resulted from the serendipitous discovery that a tyrosine kinase substrate, subsequently named caveolin, localizes to caveolae [9]. Caveolin is a 22 kD integral membrane protein with a single long hydrophobic domain that binds cholesterol and is inserted as a hairpin loop into the cytoplasmic leaflet of the lipid bilayer. Oligomerization of caveolin is believed to drive curvature formation and inward invagination of the membrane to form the pockets recognized as caveolae. Mammals encode three caveolins: caveolin 3 (CAV3) is specifically expressed in muscle cells, and the more ubiquitously expressed caveolin-1 and -2 (CAV1 and CAV2). CAV1 is essential for caveolae formation in non-muscle cells, and its expression in cells lacking the protein induces the formation of caveolae [5]. However, caveolin alone is not sufficient to form caveolae, and subsequent studies identified a second class of soluble caveolin binding partners, the cavins. Cavins are cytosolic components of the caveolae coat required to form morphologically identifiable caveolae [10]–[12]. The four cavin isoforms exhibit differential tissue-specific expression patterns; cavin-4, like CAV3, is specific to striated muscle. Cavin-1, like CAV1, is more ubiquitously expressed and is essential for the formation of caveolae. Cavin-2 disrupts caveolae formation in some tissues, but not others [12]. Cavin-3 appears not to be required for caveolae formation, but likely regulates different aspects of caveolae function. Other caveolae-associated factors have been identified on the basis of their interactions with caveolin, including the EH-domain containing protein, EHD2, which was shown to negatively regulate caveolae internalization [13],[14].

Cavins dissociate from caveolae during their purification and thus had not previously been identified as constituents of the caveolae coat. This loose association has also prevented biochemical determination of the stoichiometry and composition of the caveolae coat. In this issue of *PLOS Biology*, to solve this problem, Ludwig et al., used membrane-permeable crosslinking agents to stabilize interactions among caveolae coat constituents, isolate them following detergent solubilization by sucrose gradient centrifugation and/or immunoisolation, and then to define their stoichiometry. Cavin-1 trimers were thus identified as a core constituent of the coat. These in turn interact with ∼12 caveolin molecules and one molecule of either cavin-2 or cavin-3, which appear to compete with each other for binding to cavin-1. This basic unit oligomerizes to generate the caveolae coat. As the number of caveolin molecules per caveolae has been estimated at 140–150 [15], >10 of these core assembly units are needed to generate a complete caveolae coat. EHD2 was not associated with this coat complex. The organization of the caveolae coat is somewhat reminiscent of a clathrin coat, which has an outer shell formed by clathrin trimers and an inner shell of adaptor proteins that link clathrin to cargo molecules and the membrane [16]. As for cavin-2 and -3, there are two species of clathrin light chains that compete with each other for binding to the clathrin trimers and function, in as yet incompletely defined ways to regulate clathrin assembly and disassembly [16]. The clathrin coat has been structurally defined by EM analyses, and several investigators have applied a combination of EM approaches to begin to define the structural organization of the caveolae coat.

## Electron Microscopy Techniques to Study Caveolae Structure

Classical transmission electron microscopy (TEM) failed to detect an obvious coat-like structure on caveolae. TEM samples are chemically fixed, contrasted with heavy metals, dehydrated, and embedded in a resin before being cut into thin (50–60 nm) slices. Structures that do not resist these treatments will be lost during sample preparation. Also, the image corresponds to a two-dimensional slice through the structure of interest. When the plasma membrane was ripped off and subjected to rapid freezing and deep-etching to enable imaging of the entire structure, a coat consisting of spirals or arcs could be observed (Figure 1D) [17].

## Cryo-fixation and Electron Microscopy Tomography

**Figure 2:**
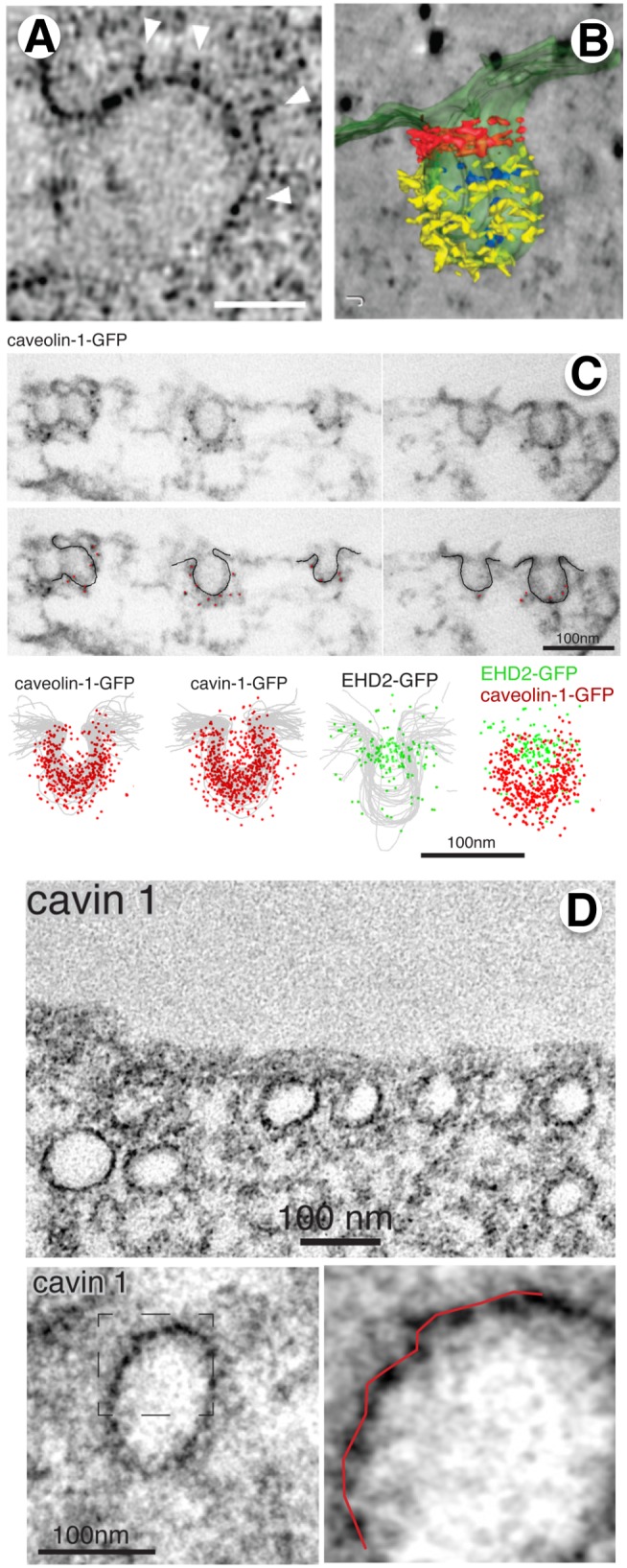
Electron microscopy tomography, immunolocalization, and the use of genetically encoded electron microscopy markers reveal high resolution ultrastructure of caveolae. (A) Thin section electron micrograph of a single caveolae in 3T3-L1 adipocyte prepared by high pressure-freezing/freeze-substitution reveals an irregular coat. Bar = 30 nm (from [18]). (B) Tomographic reconstruction of caveolae from 3T3-L1 adipocytes reveals electron dense material around the bud and neck (from [18]). (C) Pre-embedding labeling with nano-gold secondary antibodies reveals differential localization of caveolae coat components (CAV1, cavin-1) on the buds, relative to the EHD localization at the neck (from [20]). (D) Cavin-1-miniSOG fusion protein stably expressed in RPE cells allows localization of the cavin-1 protein by EM. The ∼10 nm periodicity of the DAB reaction product can be seen in the higher magnification images (from [20]).

New methods in sample preparation and EM have significantly improved the ability to preserve and detect ultrastructure and also to visualize organelles in three dimensions. These include cryo-fixation and freeze substitution, a method that avoids chemical fixation artifacts and may preserve structures better because dehydration and contrasting is done at low temperature (−50°C to −90°C). These methods of sample preparation can be coupled to EM tomography techniques that involve the use of thick sections (200–300 nm) and more powerful microscopes capable of higher acceleration voltage to view them. These thicker sections are then imaged from different angles and the images acquired automatically. Specialized software is then used to reconstruct a three-dimensional image of the organellar structures. Application of these new technologies to image caveolae in adipocytes and mouse embryo fibroblasts [18] and in epithelial cells [19] revealed a striated coat organization on caveolae bulbs (Figure 2A), as well as electron dense material in the neck region (Figure 2B). Thus, EM tomography provides high-resolution ultrastructural information; however, it cannot identify which proteins correspond to which structures.

## Immuno-electron Microscopy

EM immuno-labeling in combination with TEM can provide high-resolution insight into the localization of proteins relative to these ultrastructural features. Protein localization by TEM is commonly performed using secondary antibodies coupled to an electron dense particle such as colloidal gold of a defined size (commonly 5–20 nm). In practice, cells are embedded and cut into thin (50–60 nm) sections, and the section-surface incubated with primary- followed by, gold-coupled, secondary antibodies. However, the sensitivity of this method is limited by the bulky, and negatively charged gold particles that impair the accessibility and binding properties of the secondary antibodies. To overcome these limitations, rather than using sections of cells, Ludwig et al. selectively permeabilized whole cells with digitonin and labeled them with primary and secondary antibodies conjugated with ultra-small (<0.8 nm) gold particles before embedding and thin sectioning [20]. The nanogold clusters are detected by silver enhancement. Using this “pre-embedding” technique, they analyzed the localization of caveolin-1, cavin-1, -2, and -3, as well EHD2 on ∼50 caveolae each to create a statistical “cloud” that demarks the localization of each protein (Figure 2C). The data clearly established that cavins-1, -2, and -3 are uniformly localized throughout the bulb and excluded from the neck, EHD2 localizes predominantly to the neck.

While a powerful approach for high-resolution localization, immunoEM requires the availability of high quality, specific antibodies. Low level expression of epitope- or GFP-tagged proteins (as used by [20]) can alleviate this variable. However, the resolution of this method remains limited by the fact that the primary/secondary antibody complex measures ∼20 nm in size. This 20 nm radius makes precise location of proteins, especially relative to small structures, like membranes, difficult.

## MiniSOG, a Genetically Encoded Electron Microscopy-Compatible Tag

Both of these problems are partially mitigated by the recent development of genetically encoded tags capable of generating electron-dense signals observable by TEM. Mini singlet oxygen generator (miniSOG) is a clonable tag with only 106 amino acids, thus about half the size of GFP, consisting of a genetically engineered fluorescent flavoprotein from *Arabidopsis* phototropin 2 [21]. Upon illumination, miniSOG locally generates reactive oxygen that can catalyze the polymerization of diaminobenzidine (DAB), producing a dark precipitate that can be detected by EM [21]. Being much smaller than a primary/secondary antibody complex, miniSOG potentially allows for a more precise localization of the protein that it is directly coupled to it. However, in this case the diffusion range of the singlet oxygen and of the electron reaction products will limit resolution.

Using miniSOG fused to either cavin-1, -2, or -3, Ludwig et al. confirm their pre-embedding labeling result, and show that the cavins clearly localize to the caveolar bulb and not to the neck (Figure 2D). Importantly, because accessibility is not a limitation, miniSOG labeling is compatible with the preparation of thick (200–300 nm) sections and hence with EM tomography. Strikingly, under these conditions, miniSOG reveals a specific pattern that consists of regularly spaced peaks of electron-densities of the DAB reaction product with a periodicity of ∼10 nm. Thus, miniSOG appears to reveal protein localization, in the context of organellar ultrastructure at low nm resolution.

No technique is perfect, and a potential limitation of miniSOG technology is that the precipitate can mask the true underlying structure. It should be noted, however, that all other EM methods used to date to image caveolae suffer from this limitation in that the samples are contrasted with heavy metals that facilitate imaging but may mask the native structure. This provides a reasonable explanation as to why the caveolar coat may look different depending on the study/embedding method. Overcoming this limitation will require application of new and powerful cryo-electron tomography techniques to image samples in a closer to native state.

## Integrated Electron Microscopy: Super-Duper Resolution Microscopy

The foundation of modern cell biology still rests on observations of cellular and organellar architecture as revealed by EM. Understanding the function of complex cellular machines requires high-resolution knowledge of the spatial organization of the component parts, in the context of the underlying cellular ultrastructure. New and emerging methods in SUPER resolution light microscopy have, understandably, generated much excitement because they can provide information as to the former, at resolutions in the 20–50 nm range [22]. However, no light microscope can provide high-resolution insight into cellular ultrastructure. Combining the two through correlative light and electron microscopy (CLEM) is a powerful, but as yet, tedious approach (reviewed by [23]). The new (and old) EM methods described here are relatively routine and have become increasingly accessible. Indeed new, automated EM technologies, such as serial block face- or focused ion beam scanning EM [24]–[26] have the promise of revolutionizing EM. When applied in an integrative manner these EM technologies remain essential tools of modern cell biology.

## Abbreviations

CCV: clathrin coated vesicle
EM: electron microscopy
miniSOG: mini singlet oxygen generator
TEM: transmission electron microscopy
